# Where are we going with sentinel nodes mapping in ovarian cancer?

**DOI:** 10.3389/fonc.2022.999749

**Published:** 2022-11-03

**Authors:** Nirmala Chandralega Kampan, Chew Kah Teik, Mohammed Nasir Shafiee

**Affiliations:** Gynae-oncology Unit, Department of Obstetrics & Gynaecology, Faculty of Medicine, Universiti, Kebangsaan Malaysia, Kuala Lumpur, Malaysia

**Keywords:** sentinel lymph node, ovarian cancer, lymphadenectomy, low-volume metastases, sentinel lymph node biopsy, sentinel lymph node mapping

## Abstract

Lymph node involvement is a major predictive indicator in early-stage epithelial ovarian cancer (EOC). There is presently no effective way to determine lymph node involvement other than surgical staging. As a result, traditional ovarian cancer surgery still includes pelvic and paraaortic lymphadenectomy. However, it might be linked to higher blood loss, lengthier operations, and longer hospital stays. The creation of a technique for accurately predicting nodal status without significant lymphadenectomy is thus the subject of ongoing research. Sentinel lymph nodes (SLN) mapping is a routine procedure in oncological surgery and has been proven to be effective and safe in cervical, vulvar, and uterine cancer. On the other hand, SLN mapping is not yet widely accepted and recognized in EOC. A thorough search of the literature was conducted between January 1995 to March 2022, using PubMed and Embase. This review included studies on lymphatic outflow of the ovaries and the sentinel lymph node method. A total of 13 studies involving 212 patients who underwent sentinel lymph node mapping for ovaries were included. Both open and laparoscopic approach are used. The most popular injection site is the ovarian ligaments, and a variety of agents are utilized, although the main markers were, technetium-99m radiocolloid (Tc-99m) or indocyanine green, either alone or in combination. Overall detection rate for SLN in ovaries is 84.5% (interquartile range: 27-100%). We suggest a standardized method for sentinel lymph node mapping in ovarian cancer. The detection rates, characterization and true positive rates of the approach in investigations support further study. The use of ultra-staging is essential for lower-volume metastasis and reproducibility. To ascertain the clinical utility of sentinel node in early ovarian cancer, larger collaborative prospective clinical trials are necessary.

## Introduction

Epithelial ovarian cancer (EOC) is the most common ovarian malignancy and is the leading cause of death from gynecological cancers worldwide as up to two-thirds are detected late in advanced stages (1). The European Organization for Research and Treatment of Cancer – Adjuvant ChemoTherapy in Ovarian Neoplasm (EORTC-ACTION) – trial demonstrated that optimal surgical staging surgery was significantly associated with a superior recurrence-free and overall survival in early stages EOC in a ten-year follow-up. In addition, adjuvant chemotherapy appeared only advantageous in those with unidentified residual disease due to absent or incomplete staging surgery.

The standard of treatment consists of comprehensive surgical debulking and platinum-based chemotherapy. About a third of epithelial ovarian cancer presents in an early stage (Stage I-II) (2), enabling optimal surgical staging procedure, which includes total abdominal hysterectomy with bilateral salpingo-oophorectomy, omentectomy, peritoneal biopsies, and a pelvic and para-aortic lymph node dissection, enabling valid prognosis prediction, determines adjuvant treatment.

A midline laparotomy is the recommended standard approach to surgical staging. However, in the last two decades, advances in minimally invasive surgery (MIS) have made laparoscopy and robotic surgery a feasible approach in early-stage EOC. Various studies have shown that MIS approach has shorter hospital stays, fewer peri-operative complications and improved cosmesis compared to an open surgery (3–7). A large study involving 1112 patients who underwent planned laparoscopic surgery for Stage I EOC were reported to have similar oncological outcomes with no differences in overall survival compared to open staging (8). Similarly, in a recent retrospective study (n=455) comparing the surgical and oncological outcomes of three different modalities: open, laparoscopic and robotic, found that MIS are safe, with lower rate of post-operative morbidity and no significant difference in overall survival and progression free survival (9). This is also in agreement with another retrospective study involving a total of 254 women, who had surgical staging *via* minimal invasive approaches (laparoscopic and robotic surgery), were reported to have a good 5-year progression free survival and overall survival rates at 84.0% and 93.8% respectively (10). One of the largest study to-date comparing between robotic and laparoscopy surgery for presumed Stage 1 ovarian cancer found no significant oncologic or surgical outcome differences between these modalities (11).

EOC can metastasize through three different ways: direct spread *via* intraperitoneal, lymphogenous and hematogenous (12). Lymphatic metastases of EOC mostly develop in the para-aortic and paracaval lymph nodes followed by pelvic nodes (13). Complete pelvic and para-aortic lymph node dissection hence form an essential procedure, as recommended by The International Federation of Gynecology and Obstetrics, for clinical staging purposes, although the extent of lymph node dissection may vary according to surgical institution (14). According to the International Federation of Gynecology and Obstetrics (15), lymph node metastases in EOC is classified as FIGO stage IIIC disease (14). Following a comprehensive staging procedure, patients with a FIGO stage III ovarian cancer, unlike those with FIGO stage I ovarian cancer, are required to receive adjuvant chemotherapy (16). Omission of adequate lymphadenectomy may result in underdiagnosis of a more advanced stage in up to 20% of early stages patients (16). It is therefore critical to determine nodal status for guiding adjuvant treatment.

However, routine systematic pelvic and para-aortic lymph node dissection, as demonstrated by The Lymphadenectomy in Ovarian Neoplasms (LION) trial did not show any survival benefit even in advanced ovarian cancers (12). In addition, in early stage (stage 1-II) disease, only a minority of women would benefit from routine lymphadenectomy as the mean incidence of lymph node involvement is low at 14.2% (range 6.1-29.6%) (17), while enduring intra- and post-operative morbidities such as nerve and vessel injury, prolonged hospital stays, longer operative time, higher blood loss, need for blood transfusion and development of lymphocyst and lymphedema (16). Complete omission of lymph nodes in clinical early-stage EOC is also a concern as lymph node metastasis was found to be in greater rate in poorly differentiated (grade III) tumors (20%) and serous subtypes (23%) than in grade I (4%) and mucinous tumors (3%) (18–20).

In EOC, detection of lymph nodes metastases using radiological techniques (computed tomography (CT) scan, magnetic resonance imaging (MRI), and positron emission tomography (PET)) alone are inadequate; the sensitivity and specificity for detection of lymph node metastases with PET scan are 73.2 percent and 96.7 percent, respectively, with CT scan 42.6 percent and 95.0 percent, and MRI 54.7 percent and 88.3 percent (21).

The sentinel lymph node (SLN) is the first node or group of nodes in the lymphatic basin receiving primary lymphatic flow from tumor site, hence most likely receiving metastasis first. The SLN method involves injecting a dye or tracer into (or close to) the organ, mapping the organ’s lymphatic distribution to identify the SLN, removing it, and then examining for metastatic disease. The concept of sentinel lymph node mapping in ovarian cancer may not be useful in late disease but would be ideal in early stages ovarian cancer (Stage I-II).

The notion of sentinel lymph node mapping was first tested in patients with normal ovaries or those who had ovarian cyst surgery (22). Sentinel lymph nodes, which are typically found in the para-aortic or pelvic and para-aortic sites, are detected by lymphoscintigraphy at a mean interval of 4-6 hours following injection of radiotracer into meso-ovarian tissues (22). With the widespread use of sentinel node biopsy, enough evidence has been gathered to show that successive lymphatic propagation and tumor cell trapping in first draining lymph nodes occurs.

The concept of the sentinel node is based on the Halsted theory, which emphasizes the need of locoregional cancer treatment due to the step-wise spread of cancer (23). The foundations of sentinel node biopsy are the occurrence of an orderly and predictable pattern of lymphatic outflow to a regional lymph node basin and the performance of a first lymph node as an efficient filter for tumor cells (23, 24). A marker should facilitate the SLN to be identified with acceptable sensitivity and specificity when injected into a place that imitates the tumor’s lymphatic drainage. The lymphatic system is mapped to identify the SLN, which is then excised and examined for metastasis. The absence of an SLN does not imply the absence of lymph nodes. Failure to detect an SLN should be regarded as a mapping failure and should be treated with systematic lymphadenectomy.

The SLN mapping procedure has proven to be effective in breast, vulvar, cervical, and endometrial cancer, however for ovary, SLN studies are lacking. The aim of this work is to highlight the value of SLN mapping in the treatment of EOC and to summarize the latest data on its application.

## Methods

**Figure 1:**
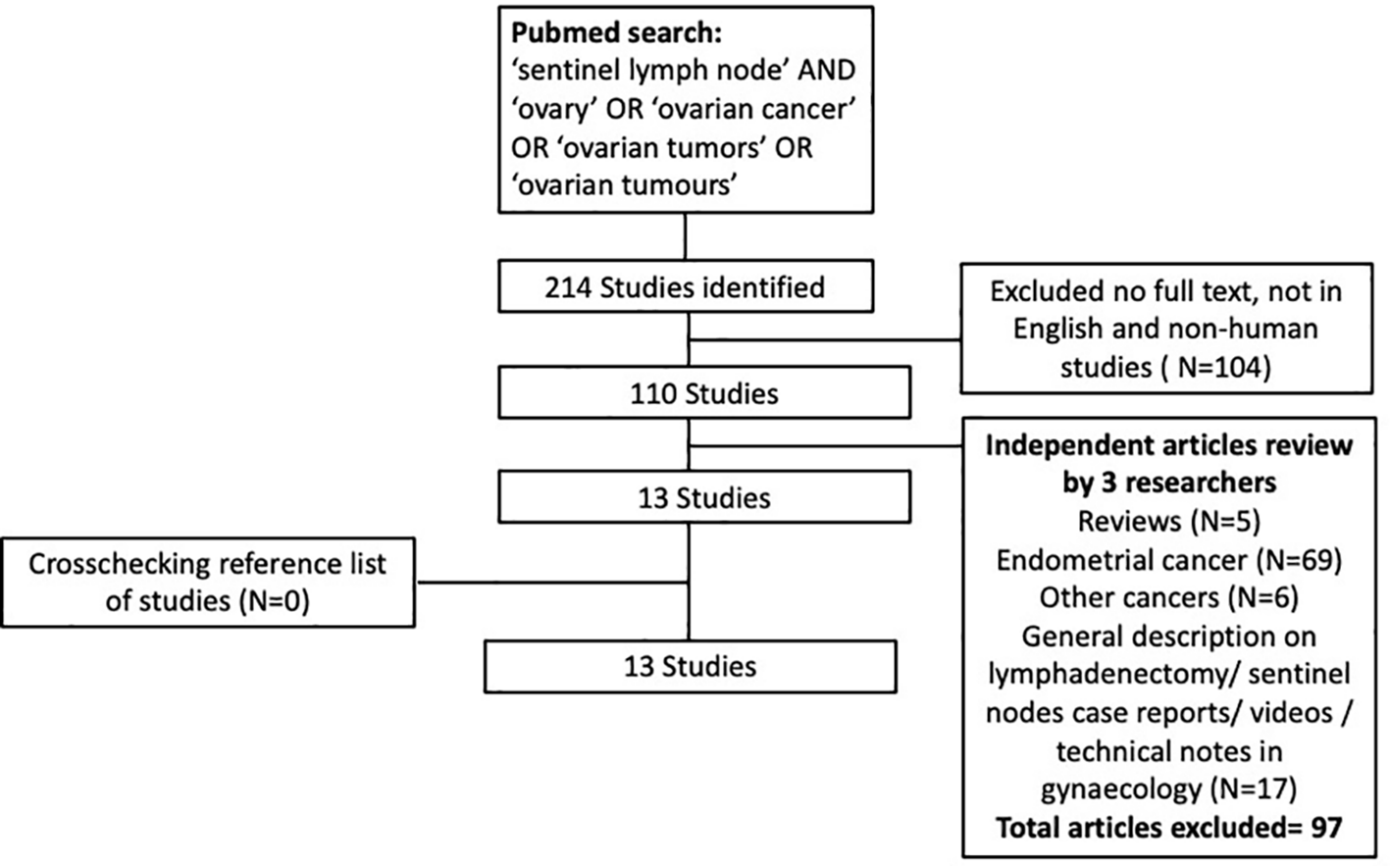
Flowchart of study selection.

This systematic review was conducted using Preferred Reporting Items for Systematic Reviews and Meta-Analyses (PRISMA) criteria (**Figure 1**). Using PubMed and Embase, a thorough systematic search of the English-language literature from January 1995 to March 2022 was conducted. All three researchers independently searched the databases and chose abstracts. The search term used included ‘sentinel lymph node’, ‘ovary’, ‘ovarian cancer’, ‘ovarian tumors’ and ‘ovarian tumors’, while search strings utilized were ‘sentinel lymph node OR sentinel node AND ovarian cancer OR ovary, ‘sentinel lymph node OR sentinel node OR ovary OR cancer’.

We incorporated primary research studies describing the SLN technique and lymphatic drainage in ovary, patients with benign or malignant ovarian mass, and patients in whom sentinel lymph node mapping of ovary irrespective of diagnosis of ovarian cancer. In addition, we also looked for other pertinent research in the reference lists of the publications identified in the initial search. Three independent reviewers separately determined whether to include titles and abstracts. To find cases of overlap, all studies from the same study group were examined. *In vitro* or cadaveric research, case reports, video articles, technical notes, review papers that did not present original data, and duplicate publications were all excluded.

The number of patients and surgical method were noted and examined as the primary indicators (open or laparoscopy). The type and dosage of tracer, site of tracer injection, the amount of time between the injection and sentinel lymph node identification, detection rate, and its location were also reported along with other surgical procedure specifics (s).

## Results

A total of 214 were initially identified, and following additional exclusion criteria (**Figure 1**), 13 studies (**Table 1**) involving a total of 212 patients who underwent sentinel lymph node mapping for ovaries were included in this review. Seven studies performed open surgery alone (16, 25–27, 30, 31, 35), while 3 studies used laparoscopic approach (22, 28, 29), and 3 studies had combined modality (32–34) including robotic surgical technique (33). A total of 131 patients (61.2%) underwent laparotomies, compared to 71(38.8%) had minimally invasive surgeries (laparoscopy n=66, converted to open=1, robotic n=4). The reasons for surgery were mainly for confirmed ovarian malignancy (Stage I-II) (29, 30, 32–34) followed by suspicion of ovarian malignancy (16, 27, 28, 34). The patients who underwent the surgery had final histology confirmation of ovarian cancer in 115 patients, followed by benign ovarian cyst (n=44), endometrial cancer (n=29), borderline ovarian mass (n=10), cervical cancer (n=3), fallopian cancer (n=1). A systematic lymphadenectomy was subsequently performed for patients with histological confirmation of ovarian cancer.

**Table 1:** Study characteristics of ovarian sentinel node mapping (N=13).

Author, Year	No of patients	Type of surgery	Histology type	Site of injection	Tracer	Tracer Dosage	Minimum waiting time after injection(min)	Sentinel lymph nodes location	Detection rate	False negative
Vanneuville et al, 1991 (22)	14	Lap	Benign ovarian cyst/tubal ligation	Mesovarium (normal ovaries)	Tc-99m + rhenium sulfide colloid	37 MBq + 0.5-0.7ml	–	Para-aortic region alone- 33% Para-aortic and pelvic 67%	85.7%	NA
Negishi et al, 2004 (25)	11	Open	Endometrial cancer (n=10), Fallopian tube cancer (n=1)	Unilateral cortex of the ovary	CH40 (charcoal solution)	0.05-0.2ml	10	Para-aortic region alone- 63% Para-aortic and pelvic 36%	100%	NA
Nyberg et al, 2011 (26)	16	Open	High-risk endometrial cancer	Hilum of ovary left (n=8), right (n=8)	Tc-99m +Blue dye	0.8 ml/2 ml	10	Above IMA- 67% Below IMA- 33%	95%	NA
Kleppe et al, 2014 (16)	21	Open	Ovarian mass (suspicious of malignant)	Ovarian ligament and the suspensory ligament, (dorsal and ventral side)	Tc-99m +Blue dye	0.5 ml/2ml	15	Para-aortic region alone- 67% Pelvic alone- 9% Para-aortic and pelvic-24%	100%	0%
Hassanzadeh et al, 2016 (27)	35	Open	Ovarian mass (suspicious of malignant)	Normal ovarian cortex (n=10) Ovarian ligament and the suspensory ligament (n=25)	Tc-99m +Blue dye (Blue dye in only 4 patients)	0.4 ml/0.4ml	10	Para-aortic region alone- 84% Pelvic alone- 8% Para-aortic and pelvic-8%	40% 84%	0% 0%
Buda et al, 2017 (28)	10	Lap	Ovarian mass (suspicious of malignant) + cervical cancer	Ovarian ligament and the suspensory ligament, (dorsal and ventral side)	ICG	0.5- 1ml (1.25 mg/ml)	Real time	Above IMA- 27% Below IMA- 53% Pelvic- 30%	90%	NA
Angelucci et al, 2016 (29)	5	Lap	Early stage ovarian cancer	Hilum of the ovary (3 right ovarian pedicle; 1 right broad ligament; 1 left ovarian parenchyma	ICG	0.5- 1ml (1.25 mg/ml)	1-3	Para-aortic region- 66% Pelvic- 34%	100%	0%
Speth et al, 2017 (30)	3	Open	Ovarian cancer (n=5, similar patient to Kleppe et al) Endometrial cancer Grade 3	Ovarian ligament and the suspensory ligament, (dorsal and ventral side)	Tc-99m + Blue dye	80 MBq + 0.2-0.5ml	15	Para-aortic region- 67% Pelvic- 33%	100%	0%
Nyberg et al, 2017 (31)	20	Open	Ovarian cancer	Mesovarium	Tc-99m + Blue dye	20 MBq + 2ml	10	Para-aortic region- 60% Pelvic- 10% Para-aortic and pelvic- 30%	100%	0%
Lago et al, 2019 (32)	10	Open (n=7) Lap (n=3)	Early stage (I-II) ovarian cancer	Ovarian ligament and the suspensory ligament stumps (deep into parametrium)	Tc-99m + ICG	37 MBq + 0.5ml	15 Detection time after injection- 54 ± 31 min (25-120)	Para-aortic region- 70% Pelvic- 87.5% Para-aortic and pelvic- 100%	100% 90% (ICG alone)	NA
Uccela et al., 2019 (SELLY) (33)	31	Lap (n=26), converted to open (n=1) Robotic (n=4) Immediate staging (n=18) Delayed staging (n=13)	Early stage (I-II) ovarian cancer	Ovarian ligament and the suspensory ligament stumps	ICG	2 ml (1.25 mg/ml)	5-20	Para-aortic region- 3.8% Pelvic- 0% Para-aortic and pelvic- 96.8%	88.9% 41.7% (second surgical staging) Overall detection rate- 21/31 = 67.7%	0%
Lago et al, 2020 SENTOV Phase II (34)	20	Open (n=11) Laparoscopy (n=9)	Ovarian mass (suspicious of malignant), n=11 Early stage (I-II) ovarian cancer, n=9	Suspensory ligament stumps and *ovarian ligament (deep into parametrium), unilateral n=19, bilateral, n=1) *- absence in one case	Tc-99m + ICG	37 MBq + 0.5ml	15 Detection time after injection- 53 ± 15 min (30-80)	Para-aortic region- 100% Pelvic- 93% Para-aortic and pelvic- 95%	100% (Tc alone) 95% (ICG alone)	NA
Laven et al, 2021 (35) (study terminated prematurely)	11	Open – primary surgery (n=8) Second surgical staging (5-8 weeks later), (n=3)	Early stage (I-IIC) ovarian cancer	Ovarian ligament (dorsal and ventral) and the suspensory ligament stumps (lateral side) Remnant of ovarian ligament	Tc-99m + Blue dye	20 MBq + 0.2 ml (15 min)	15	Para-aortic- 67% Para-aortic and pelvic-33%	27% (primary surgery) 0% (second surgery)	NA

BD, blue dye; ICG, indocyanine green; Tc-99m, technetium-99m radiocolloid.

## Injection site

The injection sites have varied, with the most preferred reported so far is into the dorsal and ventral side of ovarian ligament and suspensory ligament (n=131, 61.2%), followed by mesovarium (n=34, 15.9%), ovarium hilum (n=21, 9.81%), and ovarian cortex (n=21, 9.81%) in descending order. In four recent trials (32–35), the injection was carried out in the remaining portions of both utero-ovarian and suspensory ligament stumps and/or only into the suspensory ligament stumps according to previous hysterectomy status, either during the first surgery or a second (delayed) surgical intervention.

## Tracer agents, dose and timing

In ovarian sentinel node mapping, a variety of agents were used alone or in combination, such as technetium-99m radiocolloid (Tc-99m), patent blue, or indocyanine green (ICG). In regard to tracer Tc-99m, one study used it alone in 14 patients, while six studies (N=106) used Tc-99m in combination with blue dye (16, 26, 27, 30, 31, 35) and 2 studies (same research group) (32, 34) used it in combination with ICG (N=30). Indocyanine green was injected alone in three studies involving 46 patients (28, 29, 33).

Negishi et al. (25) is the only investigator who used 10 mg of carbon particles (diameter of 20 nm and 4 mg of polyvinyl pyrolidone with a concentration of 0.05–0.2 ml), combined in a solution of charcoal (1 ml) to trace sentinel nodes. For radiocolloid, the dosage of tracer varies between studies with highest dose at 80 MBq (30, 32, 34, 35), while for ICG, each researcher administered between 0.5–2 mL of a solution containing 1.25 mg/mL of indocyanine green, and for blue dye, the volume ranges between 0.2-2.0 ml.

The interval between tracers being injected and sentinel lymph nodes being found when Tc-99m and blue dye used together was 10 minutes in 71 patients (26, 27, 31) and 15 minutes in 35 patients (16, 30, 35). In two studies by the same research group, the minimum waiting time for radiocolloid in combination with ICG was 15 minutes before examination of area of migration, followed by commencement of sentinel node removal procedure after 30 minutes duration (32, 34). When ICG is administered alone, the waiting time is the shortest for sentinel lymph node mapping, with Buda et al, performing mapping right away after injecting the tracer laparoscopically for 10 patients (28), while in another study, the median waiting interval for 5 patients was 2 minutes (29).

## Sentinel Lymph nodes detection rate and location

Sentinel lymph nodes were discovered in 175 of 207 individuals, for a detection rate overall of 84.5% (interquartile range: 27-100%). The detection rate for radiocolloid (16, 22, 26, 27, 30, 35) (with or without blue dye; N=120) was 82.5%, while the detection rate for radiocolloid and ICG combined was 100% (32, 34) and when ICG used alone, the detection rate was 82.8% (N=76) (28, 29, 32–34). There is reported lack of tissue penetration with the use of blue dye in several studies (16, 26, 27). Blue dye was observed transperitoneally in only less than half of patients in Nyberg et al. (26), while in Kleppe et al. (16) the blue staining was not detected in transperitoneal mapping but seen in one-third of patients during retroperitoneal exploration. Therefore, the presence of sentinel nodes is not necessarily ruled out in the absence of blue staining.

The majority of patients (89.6 percent, N=186) had at least one lymph node detected following injection just beneath the peritoneum (ovarian ligaments, mesovarium, ovarian hilum). In two studies, an injection into the ovarian cortex appeared to be less receptive (71.4 percent, N=21). In the studies that distinguished between the para-aortic and pelvic regions, the sentinel lymph nodes were found in the para-aortic region only in 99 out of 181 patients (54.6%), the pelvic region only in 26 patients (14.4%), and both the para-aortic and pelvic regions in 80 patients (44.2%). Two studies (N=26) found the sentinel lymph node to be either above or below the level of the inferior mesenteric artery (28, 31). According to three studies, the majority of the SNs associated to the right ovary were discovered below the level of the inferior mesenteric artery, whereas the majority of the SNs related to the left ovary were found above the level of the inferior mesenteric artery (16, 26, 28).

While the detection rate of at least one SLN following the injection of a tracer prior to tumor resection and analysis of frozen sections were high between 87.5-100%, the detection rate of SLNs in post tumor (adnexal) resection operations varied. Four investigations evaluated the identification of SLNs following the injection of tracer into the remaining ovarian ligaments following the resection of the adnexa, either in the same surgical setting or in a subsequent surgery (32–35). While the detection rate in two studies by Lago et al. (N=30) (32, 34) was 100% using radiocolloid and ICG, and 90-95% using ICG alone, Laven et al. had the lowest detection rate at 27% (35) using radiocolloid and blue dye tracer. In studies by Lago et al. (32, 34) the researchers injected the tracer deeply into the parametrium as opposed to superficially under the peritoneum as done in Laven et al (35), resulting in 88% (32) and 93% (34) of patients with a detectable pelvic (non-para aortal) sentinel node. Conversely, three sentinel nodes in Laven et al. (35), were found all at the para-aortal/para-caval level. This may be explained by possible changes in lymphatic drainage following ovary resection, which render accurate identification of the para-aortal sentinel node following previous resection less reliable.

In patients with Stages I-II ovarian cancer who had sentinel lymph node mapping and systematic lymphadenectomy, the characteristics of sentinel lymph nodes are as listed in **Table 2**. Only eight studies provided information on the characteristics of sentinel lymph nodes. A total of 18 cases of SLN positive were observed (8.7%) out of 208 SLN removed. There is a total of 4 macrometastases (1.9%), 3 micrometastases (1.4%) and one case of isolated tumor cells (0.5%) following ultrastaging (36). The sentinel lymph node in the para-aortic field which was falsely negative in one patient in the trial by Lago et al. (34) because of absence of migration of tracer from ovarian stump sites following resection of the adnexa, was identified as macrometastasis after performing ultrastaging procedure (36).

**Table 2:** Lymph node characteristics in epithelial ovarian cancer patients after SLN mapping and systemic lymphadenectomy (N=8).

Author, Year	No of patients	SLN positive n(%)	Non-SLN positive n(%)	Type of SLN involvement	Sensitivity	Specificity	Negative Predictive Value
Hassanzadeh et al, 2016 (27)	17	4/20 (20%)	NA	NA	100%	NA	NA
Angelucci et al, 2016 (29)	5	0/7 (0%)	0.107 (0%)	NA	NA	100%	100%
Nyberg et al, 2017 (31)	4	1/8 (13%)	4/121 (3.3%)	NA	100%	NA	NA
Kleppe et al, 2014 (16)	6	3/23 (13%)	1/65 (1.5%)	3-Micro M	100%	NA	NA
Buda et al, 2017 (28)	7	0/11 (0%)	0/165 (0%)	NA	NA	100%	NA
Uccela et al., 2019 (SELLY) (33)	31	4/31 (12.9%)	0/31 (0%)	1-ITC 3-Macro M	100%	100%	100%
Lago et al, 2021 (36) Results from 2 studies-Lago et al, 2019 (32) Lago et al, 2020 SENTOV Phase II (34)	30 (10 from (32) and 20 from (34)	6/114* (5.3%) *ultra-staging	1 non SLN detected	1-Macro M 1-ITC (Ultrastaging)	NA	NA	NA
Laven et al, 2021 (35) (study terminated prematurely)	3	0/4	NA	No metastases	NA	NA	NA

ITC, isolated tumor cell; M, metastases; SLN, sentinel lymph nodes; SLN, sentinel lymph node; NA,-not available.

Only two studies (same research group) performed ultrastaging of SLN. In Lago et al. (36), a total of 30 patients underwent SLN mapping and pathological ultrastaging with a slice thickness of 200 microns to examine the function of SLN ultrastaging in early-stage ovarian cancer. The detection rates for radiocolloid and ICG were high, reaching 30/30 (100%) and 28/30 (93.3%), respectively. Following ultrastaging, six patients were upgraded: two patients identified macrometastases that had previously gone undetected, and four patients discovered implantation in other sites (omentum, fallopian tube, Douglas cavity, etc).

## Discussion

With an overall detection rate of 84.5% percent (interquartile range: 27-100%), our review demonstrates that sentinel node detection from the ovary appears to be promising. This is in agreement with a systematic review by Dell’Orto et al. which included 10 studies on ovarian sentinel nodes (37). However, the availability of limited number of studies and the total sample size of patients included are insufficient to allow for firm conclusions on the choices of tracer(s) type, timing and precise location.

In ovarian sentinel node mapping, the role apart from technetium-99m radiocolloid as the tracer of choice, indocyanine green use has been increasingly popular. Although the use of technetium-99m radiocolloid has yielded high rates of detection of sentinel nodes in various studies but it has several disadvantages. The use of radiocolloid for sentinel nodes detection are an expensive procedure, requires pre-operative preparation and professional help from nuclear medicine department, time consuming and carry risk of radioactivity. The detection rate of blue dye seems to be poor with reported lack or absence of tissue penetration in several studies (16, 26, 27). It has been reported that the success rate sentinel node mapping in uterine cancers is significantly decreased with increasing BMI irrespective of dye used, however the use of blue dye compared to ICG yield superior SLN detection rates (38). ICG, compared to blue dye, has been found to have improved tissue penetration, which enhance visibility (39, 40). A successful demonstration of the use of ICG to detect sentinel nodes was demonstrated in a recent video article (41) on a patient enrolled in SELLY trial undergoing laparotomy for a large ovarian mass with suspected malignancy. The author, Turco et al. (41) demonstrated injection of ICG of 5mg/ml with a 20-gauge spinal needle into the perivascular connective tissue surrounding infundibulopelvic ligament and utero-ovarian ligament of the affected ovary. Removal of the affected adnexal mass was performed following a waiting time of 15 minutes. Once ovarian malignancy is confirmed, the retroperitoneum area bilaterally was subsequently exposed along the Tort fascia till up to left renal vein and the sentinel nodes were detected using near-infrared fluorescent system camera (41).

The exact timing for detection of SLN is not reported in most studies. In Lago et al. pilot and Phase II studies, using a combination of radiocolloid and ICG tracer, the mean detection time for SLN was 53.3 ± 20.3 min (32, 34). In these studies, a minimum waiting time of 15 minutes were adhered to before exploration of sentinel nodes were performed (32, 34). Similarly, in majority of studies using a combination of radiocolloid and ICG, a minimum of 10 to 15 minutes of waiting time is allowed before proceeding with SLN mapping to ensure adequate travel of the tracer to the lymph nodes. When ICG is used alone, the waiting time is shorter between 5 to 20 minutes as demonstrated in SELLY trial (33). The use of ICG have also been done in real time by Buda et al. (28) and with only 1 to 3 min waiting time in some studies (29).

Currently, injections made to ovarian cortex is avoided as it has the worst detectability and is associated with risk of tumor dissemination (27, 40). Majority of studies found that the ovarian suspensory ligament and/or ovarian ligaments are a safe and repeatable option when it comes to injection sites, hence this is recommended. Studies employing this route had high detection rate of SLN ranging between 84 to 100% when performed prior to tumor removal. In Lago et al, both radiocolloid and ICG tracers were used simultaneously following removal of adnexal mass in 15 of their patients (34). Injections points were at infundibulopelvic and utero-ovarian ligament stumps either unilaterally or bilaterally. A 27G needle was used to inject saline solution (0.2ml) containing 37 mBq radiocolloid and 0.5ml ICG (1.25mg/ml) simultaneously. This technique yielded a high detection of sentinel node at 95% in the pelvic and para-aortic regions. The advantage of this technique according to the author is that it can be performed in both open and laparoscopic surgery (32, 34). This sentinel node mapping technique is also applicable and can be done after the removal of the primary tumor, therefore limiting the use of 99mTc and ICG to only those cases where malignancy has been proven. The drawback of this procedure is in its amplified length of surgery to an extra one hour (32, 34), which may increase operational cost and higher anesthesia risk. Various studies discourage from multiple sites of tracer injection as this can lead to tracer spillage in the retroperitoneal region, leading to poor visibility and lower detection rates of sentinel nodes (31, 33). Adequate training of surgeon is also required to acquire a good technique for tracer injection with avoidance of dye extravasation.

Although studies by Lago et al. (32, 42) have shown that it is feasible to perform SLN detection following injection of tracer into the remnants of both ovarian ligaments after excision of ovarian tumor with high detection rate (95-100%), two other studies only demonstrated a low percentage of SLN detection rate at 41.7% and 27% (33, 35). A difference in the technique of tracer injection may have resulted in a dissimilar outcome between these three studies, for example in Lago et al. (32, 34), the ICG was injected deeply into the parametrium as opposed to superficial injection under the peritoneum in Laven et al. (35). It was postulated that deep parametrium injection by Lago et al. may have resulted in detection of uterine rather than ovarian pelvic lymphatic drainage. However, the detection rate of sentinel nodes remained of comparable yield to previous studies. A potential explanation maybe that the lymphatic drainage continues in bi-directional pathway from the infundibulopelvic and utero-ovarian stumps to the para-aortic and pelvic fields, respectively, meaning that the ovary excision has no initial effect on the tracer’s drainage. Following ovarian tumor removal, continuous lymphatic perfusion from the original organ begin to cease, resulting in gradual obstruction and deterioration of the lymphatic drainage pathway. This may explain a higher positive rate of sentinel nodes (88.9% vs. 41.7%) found within patients with immediate surgical staging than with delayed surgical staging (33). In addition, the SLN mapping was not well localized when the SLN procedure was delayed 5–8 weeks after tumor resection (35).

A thorough understanding of the lymphatic pathways, hence is necessary to optimize the sentinel node procedure in ovarian cancer. Studies detailing the lymphatic drainage routes for ovarian tumors are sparse. Lymphatic metastases of EOC mainly occurs in the para-aortic and paracaval regions followed by pelvic lymph nodes (16, 24, 25, 40). According to Kleppe et al. (24), the sentinel lymph node in ovarian cancer is found in the para-aortic and paracaval regions, obturator fossa and surrounding internal iliac arteries, and inguinal regions. Kleppe et al. (24), used immunohistochemical analysis from a microscopic perspective to identify that the ovaries have two major and one minor lymphatic drainage channel (24). The first channel is abdominal pathway travelling from the ovaries to the para-aortic and paracaval lymph nodes *via* the suspensory ligament (infundibulopelvic ligament) (16, 24), while another channel is the pelvic pathway, running along ovarian ligament to obturator fossa and the internal iliac artery (16, 24, 40) and a third minor channel to the inguinal lymph nodes *via* the round ligament.

In a recent study, the lymphatic outflow following administration of ICG was demonstrated in a video (43). The lymphatic drainage of the ovary and the uterine corpus are found to be identical, using the same lymphatic pathways down the ovarian vessels to the right and left infrarenal, paraaortic regions, and the pelvic pathway along the uterine artery to the inter-iliac region. In addition, the two main routes along the Müllerian (uterine) and mesonephric (ovarian) pathways can be demonstrated reproducibly in terms of intraoperative dynamics of ICG drainage (43). Various studies support the notion that the para-aortic region serves as the primary lymphatic drainage pathway from the ovary, with the left ovary’s sentinel nodes often being positioned higher than those of the right ovary. In addition, it appears that the pelvic sentinel nodes associated with the left ovary are situated higher in the pelvis than those associated with the right ovary (25, 31–33). Hence, the ovary is far more difficult to approach than the vulvar, cervix, or endometrial cancer, where the injection site is easily accessible prior to surgical preparation. Along with the difficulties of knowing where, when, and what to inject, there is the unfavorable procedural step of having to remove the clinically suspect ovary before determining whether it is malignant, necessitating the performance of sentinel node mapping. A larger multicenter investigation should assess the validity of the SN concept in ovarian cancer and its clinical applications.

We propose the use of radiocolloid in combination with ICG (where the fluorometric imaging technology is available) with a median 15-minute interval as it has an acceptable SLN detection rate. Uccella et al., currently the largest prospective study, SELLY published on SLN in ovarian cancer (N=31), use ICG alone as their tracer laparoscopically. The use of indocyanine green (ICG) unlike radiocolloid technetium, does not necessitate pre-operative planning and availability of nuclear medicine facility and can be administered intra-operatively (33). SELLY trial (NCT03452982) is still actively recruiting a larger participant and the result of this trial is much awaited to further encourage the use of ICG alone for SLN detection. A large single-center prospective trial (NCT02997553) involving 744 participants comparing between ICG alone and in combination with radiocolloid may also help strengthen the role of ICG in SLN detection. In sentinel node mapping for ovarian tumors, we also propose ovarian suspensory ligament and/or ovarian ligaments as a safe and repeatable site for tracer injection with avoidance of multiple injection spots to reduce dye leakage.

Many surgeons will find it an inconvenient procedural step of having to remove a clinically suspect ovary before it is confirmed to be malignant, necessitating sentinel node mapping, however there is currently insufficient evidence to recommend SLN detection post ovarian tumor resection. It is also postulated that changes to lymphatic drainage post tumor resection might lead to artificially increased detection rates. Future research is needed to study anatomical information on the potential paths used by the ovarian lymphatic drainage following ovarian tumor resection which may help to explain differences in SLN detection.

There is currently no recommendation from NCCN Clinical Practice Guidelines in Oncology (2022 edition) for application of SLN technique in ovarian cancer. The surgical norm for early-stage ovarian cancer is a (mid-line) laparotomy, in accordance with ovarian cancer surgery recommendations from European Society of Gynaecological Oncology (ESGO). Laparotomy should therefore be the first option, especially when there are significant ovarian tumors present, for sentinel lymph node mapping. These same recommendations state that laparoscopic surgery for sentinel lymph node mapping should only be considered when a second procedure is required to confirm the disease’s stage or when there are small, suspicious ovarian nodules present. In both open and laparoscopic approach, Lago et al. (32, 34) and Uccella et al. (33), found no intraoperative complications, or 30-day side effects associated with the use of 99mTc or ICG, with overall complication rate low at 9.6% in SELLY’s trial (33).

The use of laparoscopic as a modality may also be technically challenging and require a longer learning curve. This includes significant chance of protocol breaches, procedure abandonment and inadvertent injection of ICG tracer into non-targeted areas as seen in SELLY’s trial (33). In order to prevent accidental ICG spillage in the trocar and subsequent tracer spillage throughout the entire operating field, a few suggestions have been made by Uccella et al. (33), including using transcutaneous needle insertion rather than using laparoscopic needles through trocars, using laparoscopic forceps to guide the needle to the ovarian pedicle, aspirating while retracting the needle from the ovarian pedicle to prevent tracer spillage, and finally getting the laparoscopic camera close to the lymphatic tissue to enable better definition of lymphatic drainage and easier identification of SLN. In Lago et al, dye spillage is prevented by placement of clamps at the site of tracer injection after completion of administration (32).

The use of ultrastaging is essential for lower-volume metastasis detection and to provide reproducible information between upcoming studies, as evidence about SLN in ovarian cancer is growing. There is a chance of getting false-negative results for isolated tumor cells and micrometastases if ultrastaging SLN processing is not performed as demonstrated by Lago et al. (36). In this study, the sentinel nodes were additionally incised perpendicular to the maximum diameter of the node into a thin section of approximately 2 mm (bread-loaf slicing technique). The nodes were subjected to standard H&E staining and serial examination of levels up to 6 levels, with depths increasing in 200 µm increments until reaching the bottom of the sample (36). Immunostaining with immunohistochemical staining for CK AE1/3 was included in the presence of macrometastasis (36). Following ultrastaging, the diameter of one pelvic SLN’s metastatic implant shifted to macrometastasis (36). A uniform protocol for ultrastaging is therefore essential for lower-volume metastasis detection and to provide reproducible information between upcoming studies, as evidence about SLN in ovarian cancer is growing.

## Conclusions

The reported experience of SLN in ovarian cancer is restricted to a few studies with a small patient sample. However, there is growing support for its feasibility, and its acceptable negative predictive value. However, further evidence from phase III clinical studies is required to clarify the true negative predictive value, critically regarding patient safety. There is lack of studies on the characterization and accuracy of sentinel nodes in detecting metastases in early ovarian cancer. To ascertain the sentinel node technique’s negative predictive value and better characterize its clinical utility in early ovarian cancer, a larger collaborative clinical investigation will be necessary.

## Data availability statement

The original contributions presented in the study are included in the article. Further inquiries can be directed to the corresponding author.

## Author contributions

NK conceived the topic and wrote the first draft. CK and MS reviewed the manuscript, tables, and images. All authors contributed to the article and approved the submitted version.

## Funding

We received support from Dana Perdana Impak Fund, DIP-2021-016 from Centre for Research and Instrumentation Management, Universiti Kebangsaan Malaysia.

## Conflict of interest

The authors declare that the research was conducted in the absence of any commercial or financial relationships that could be construed as a potential conflict of interest.

## Publisher’s note

All claims expressed in this article are solely those of the authors and do not necessarily represent those of their affiliated organizations, or those of the publisher, the editors and the reviewers. Any product that may be evaluated in this article, or claim that may be made by its manufacturer, is not guaranteed or endorsed by the publisher.

## References

[1] Sung H Ferlay J Siegel RL Laversanne M Soerjomataram I Jemal A . Global cancer statistics 2020: GLOBOCAN estimates of incidence and mortality worldwide for 36 cancers in 185 countries. CA Cancer J Clin (2021) 71(3):209–49. doi: 10.3322/caac.21660 33538338

[2] Holschneider CH Berek JS . Ovarian cancer: Epidemiology, biology, and prognostic factors. Semin Surg Oncol (2000) 19(1):3–10. doi: 10.1002/1098-2388(200007/08)19:1<3::AID-SSU2>3.0.CO;2-S 10883018

[3] Tuschy B Berlit S Brade J Sütterlin M Hornemann A . Gynaecological laparoscopic surgery for benign conditions: do women care about incisions? Eur J Obstet Gynecol Reprod Biol (2013) 169(1):84–7. doi: 10.1016/j.ejogrb.2013.02.002 23474383

[4] Borahay MA Tapısız ÖL Alanbay İ Kılıç GS . Outcomes of robotic, laparoscopic, and open hysterectomy for benign conditions in obese patients. J Turkish German Gynecol Assoc (2018) 19(2):72. doi: 10.4274/jtgga.2018.0018 PMC5994808 29699956

[5] Bouquet de Joliniere J Librino A Dubuisson J-B Khomsi F Ben Ali N Fadhlaoui A . Robotic surgery in gynecology. Front Surg (2016) 3:26. doi: 10.3389/fsurg.2016.00026 27200358 PMC4852174

[6] Levy L Tsaltas J . Recent advances in benign gynecological laparoscopic surgery. Faculty Rev (2021) 10:10–60. doi: 10.12703/r/10-60 PMC8361750 34409423

[7] Gitas G Hanker L Rody A Ackermann J Alkatout I . Robotic surgery in gynecology: Is the future already here? Minimally Invasive Ther Allied Technol (2022) 31:6, 815–824. doi: 10.1080/13645706.2021.2010763 34989636

[8] Melamed A Keating NL Clemmer JT Bregar AJ Wright JD Boruta DM . Laparoscopic staging for apparent stage I epithelial ovarian cancer. Am J Obstet Gynecol (2017) 216(1):50. e1–50. e12. doi: 10.1016/j.ajog.2016.08.030 27567562 PMC5618712

[9] Cianci S Capozzi VA Rosati A Rumolo V Corrado G Uccella S . Different surgical approaches for early-stage ovarian cancer staging. a Large monocentric experience. Front Med (2022) 9. doi: 10.3389/fmed.2022.880681 PMC9081786 35547212

[10] Gallotta V Jeong SY Conte C Trozzi R Cappuccio S Moroni R . Minimally invasive surgical staging for early stage ovarian cancer: a long-term follow up. Eur J Surg Oncol (2021) 47(7):1698–704. doi: 10.1016/j.ejso.2021.01.033 33573854

[11] Gallotta V Cicero C Conte C Vizzielli G Petrillo M Fagotti A . Robotic versus laparoscopic staging for early ovarian cancer: A case-matched control study. J Minim Invasive Gynecol (2017) 24(2):293–8. doi: 10.1016/j.jmig.2016.11.004 27856387

[12] Deng T Huang Q Wan T Luo X Feng Y Huang H . The impact of lymph node dissection on survival in patients with clinical early-stage ovarian cancer. J Gynecol Oncol (2021) 32(3):1–10. doi: 10.3802/jgo.2021.32.e40 PMC8039180 33825356

[13] Burghardt E Girardi F Lahousen M Tamussino K Stettner H . Patterns of pelvic and paraaortic lymph node involvement in ovarian cancer. Gynecol Oncol (1991) 40(2):103–6. doi: 10.1016/0090-8258(91)90099-Q 2010101

[14] Berek JS Renz M Kehoe S Kumar L Friedlander M . Cancer of the ovary, fallopian tube, and peritoneum: 2021 update. Int J Gynaecol Obstet (2021) 155 Suppl 1:61–85. doi: 10.1002/ijgo.13878 34669199 PMC9298325

[15] Prat J FIGO Committee on Gynecologic Oncology . Staging classification for cancer of the ovary, fallopian tube, and peritoneum. Int J Gynaecol Obstet (2014) 124(1):1–5. doi: 10.1016/j.ijgo.2013.10.001 24219974

[16] Kleppe M Brans B Van Gorp T Slangen BF Kruse AJ Pooters IN . The detection of sentinel nodes in ovarian cancer: a feasibility study. J Nucl Med (2014) 55(11):1799–804. doi: 10.2967/jnumed.114.144329 25332439

[17] Bogani G Ditto A Pinelli C Lopez S Chiappa V Raspagliesi F . Ten-year follow-up study of long-term outcomes after conservative surgery for early-stage ovarian cancer. Int J Gynaecol Obstet (2020) 150(2):169–76. doi: 10.1002/ijgo.13199 32415982

[18] Kleppe M Wang T Van Gorp T Slangen BF Kruse AJ Kruitwagen RF . Lymph node metastasis in stages I and II ovarian cancer: a review. Gynecol Oncol (2011) 123(3):610–4. doi: 10.1016/j.ygyno.2011.09.013 21982047

[19] Bogani G Tagliabue E Ditto A Signorelli M Martinelli F Casarin J . Assessing the risk of pelvic and para-aortic nodal involvement in apparent early-stage ovarian cancer: A predictors- and nomogram-based analyses. Gynecol Oncol (2017) 147(1):61–5. doi: 10.1016/j.ygyno.2017.07.139 28779965

[20] van de Vorst R Hoogendam JP van der Aa MA Witteveen PO Zweemer RP Gerestein CG . The attributive value of comprehensive surgical staging in clinically early-stage epithelial ovarian carcinoma: A systematic review and meta-analysis. Gynecol Oncol (2021) 161(3):876–83. doi: 10.1016/j.ygyno.2021.04.007 33849726

[21] Yuan Y Gu ZX Tao XF Liu SY . Computer tomography, magnetic resonance imaging, and positron emission tomography or positron emission tomography/computer tomography for detection of metastatic lymph nodes in patients with ovarian cancer: a meta-analysis. Eur J Radiol (2012) 81(5):1002–6. doi: 10.1016/j.ejrad.2011.01.112 21349672

[22] Vanneuville G Lebouedec G Mestas D Scheye T Dauplat J Veyre A . Functional aspects of lymphatic drainage of the human ovary in vivo explored with isotopic lymphography. Bull L’association Des Anatomistes (1991) 75(229):177–9. 1777710

[23] Tanis PJ Nieweg OE Valdés Olmos RA Th Rutgers EJ Kroon BBR . History of sentinel node and validation of the technique. Breast Cancer Res (2001) 3(2):109. doi: 10.1186/bcr281 11250756 PMC139441

[24] Kleppe M Kraima AC Kruitwagen RF Van Gorp T Smit NN van Munsteren JC . Understanding lymphatic drainage pathways of the ovaries to predict sites for sentinel nodes in ovarian cancer. Int J Gynecol Cancer (2015) 25(8):1405–14. doi: 10.1097/IGC.0000000000000514 PMC5106084 26397066

[25] Negishi H Takeda M Fujimoto T Todo Y Ebina Y Watari H . Lymphatic mapping and sentinel node identification as related to the primary sites of lymph node metastasis in early stage ovarian cancer. Gynecol Oncol (2004) 94(1):161–6. doi: 10.1016/j.ygyno.2004.04.023 15262135

[26] Nyberg RH Korkola P Mäenpää J . Ovarian sentinel node: is it feasible? Int J Gynecol Cancer (2011) 21(3):568–72. doi: 10.1097/IGC.0b013e318211ef75 21436705

[27] Hassanzadeh M Hosseini Farahabadi E Yousefi Z Kadkhodayan S Zarifmahmoudi L Sadeghi R . Lymphatic mapping and sentinel node biopsy in ovarian tumors: A study using intra-operative Tc-99m-Phytate and lymphoscintigraphy imaging. J Ovarian Res (2016) 9(1):55. doi: 10.1186/s13048-016-0265-4 27604260 PMC5013627

[28] Buda A Passoni P Corrado G Bussi B Cutillo G Magni S . Near-infrared fluorescence-guided sentinel node mapping of the ovary with indocyanine green in a minimally invasive setting: A feasible study. J Minim Invasive Gynecol (2017) 24(1):165–70. doi: 10.1016/j.jmig.2016.09.006 27670732

[29] Angelucci M Corrado G Mancini E Baiocco E Chiofalo B Zampa A . Laparoscopic indocyanine green sentinel lymph node mapping in early ovarian cancer. a pilot study and review of the literature. Ital J Gynaecol Obstet (2016) 28(5):23–8. doi: 10.14660/2385-0868-56

[30] Speth SC Kruitwagen RF Kleppe M Pooters IN Van Gorp T Slangen BF . Comparison of intraoperative γ-probe imaging and postoperative SPECT/CT in detection of sentinel nodes related to the ovary. J Nucl Med (2017) 58(2):243–5. doi: 10.2967/jnumed.116.183426 27738006

[31] Nyberg RH Korkola P Mäenpää JU . Sentinel node and ovarian tumors: A series of 20 patients. Int J Gynecol Cancer (2017) 27(4):684–9. doi: 10.1097/IGC.0000000000000948 28375928

[32] Lago V Bello P Montero B Matute L Padilla-Iserte P Lopez S . Clinical application of the sentinel lymph node technique in early ovarian cancer: a pilot study. Int J Gynecol Cancer (2019) 29(2):377–81. doi: 10.1136/ijgc-2018-000049 30718316

[33] Uccella S Nero C Vizza E Vargiu V Corrado G Bizzarri N . Sentinel-node biopsy in early-stage ovarian cancer: preliminary results of a prospective multicentre study (SELLY). Am J Obstet Gynecol (2019) 221(4):324.e1–324.e10. doi: 10.1016/j.ajog.2019.05.005 31082385

[34] Lago V Bello P Montero B Matute L Padilla-Iserte P Lopez S . Sentinel lymph node technique in early-stage ovarian cancer (SENTOV): a phase II clinical trial. Int J Gynecol Cancer (2020) 30(9):1390–6. doi: 10.1136/ijgc-2020-001289 PMC7497563 32448808

[35] Laven P Kruitwagen R Zusterzeel P Slangen B Van Gorp T van der Pol J . Sentinel lymph node identification in early stage ovarian cancer: is it still possible after prior tumor resection? J Ovarian Res (2021) 14(1):1–6. doi: 10.1186/s13048-021-00887-w 34645514 PMC8513191

[36] Lago V Montero B López S Padilla-Iserte P Matute L Marina T . Ultrastaging protocol in sentinel lymph node for apparent early stage ovarian cancer. Gynecol Oncol (2021) 161(2):408–13. doi: 10.1016/j.ygyno.2021.03.001 33712275

[37] Dell'Orto F Laven P Delle Marchette M Lambrechts S Kruitwagen R Buda A . Feasibility of sentinel lymph node mapping of the ovary: a systematic review. Int J Gynecol Cancer (2019) 29(7):1209–15. doi: 10.1136/ijgc-2019-000606 31474589

[38] Eriksson AG Montovano M Beavis A Soslow RA Zhou Q Abu-Rustum NR . Impact of obesity on sentinel lymph node mapping in patients with newly diagnosed uterine cancer undergoing robotic surgery. Ann Surg Oncol (2016) 23(8):2522–8. doi: 10.1245/s10434-016-5134-2 PMC4929032 26905542

[39] Hackethal A Hirschburger M Eicker SO Mücke T Lindner C Buchweitz O . Role of indocyanine green in fluorescence imaging with near-infrared light to identify sentinel lymph nodes, lymphatic vessels and pathways prior to surgery - a critical evaluation of options. Geburtshilfe Frauenheilkd (2018) 78(1):54–62. doi: 10.1055/s-0043-123937 29375146 PMC5778195

[40] Wang T Xu Y Shao W Wang C . Sentinel lymph node mapping: Current applications and future perspectives in gynecology malignant tumors. Front Med (2022) 9. doi: 10.3389/fmed.2022.922585 PMC9276931 35847801

[41] Turco LC Vargiu V Nero C Fagotti A Scambia G Cosentino F . Laparotomy approach to sentinel lymph node detection in ovarian cancer using a near-infrared fluorescent system camera with indocyanine green dye. Int J Gynecol Cancer (2020) 30(5):712–3. doi: 10.1136/ijgc-2019-001110 32079713

[42] Lago V Bello P Matute L Padilla-Iserte P Marina T Agudelo M . Sentinel lymph node technique in apparent early ovarian cancer: Laparoscopic technique. J Minim Invasive Gynecol (2020) 27(5):1019–20. doi: 10.1016/j.jmig.2019.09.790 31628986

[43] Abu-Rustum NR Angioli R Bailey AE Broach V Buda A Coriddi MR . IGCS intraoperative technology taskforce. update on near infrared imaging technology: beyond white light and the naked eye, indocyanine green and near infrared technology in the treatment of gynecologic cancers. Int J Gynecol Cancer (2020) 30(5):670–83. doi: 10.1136/ijgc-2019-001127 PMC8867216 32234846

